# The role of SARS-CoV-2 N protein in diagnosis and vaccination in the context of emerging variants: present status and prospects

**DOI:** 10.3389/fmicb.2023.1217567

**Published:** 2023-08-14

**Authors:** Wanchen Song, Zhongbiao Fang, Feike Ma, Jiaxuan Li, Zhiwei Huang, Yanjun Zhang, Jianhua Li, Keda Chen

**Affiliations:** ^1^School of Medical Technology and Information Engineering, Zhejiang Chinese Medical University, Hangzhou, China; ^2^Shulan International Medical College, Zhejiang Shuren University, Hangzhou, China; ^3^School of Laboratory Medicine and Life Sciences, Wenzhou Medical University, Wenzhou, China; ^4^Key Laboratory of Public Health Detection and Etiological Research of Zhejiang Province, Department of Microbiology, Zhejiang Provincial Center for Disease Control and Prevention, Hangzhou, China

**Keywords:** SARS-CoV-2, nucleocapsid protein, diagnostic methods, vaccine development, variants of concern, immune escape

## Abstract

Despite many countries rapidly revising their strategies to prevent contagions, the number of people infected with Severe acute respiratory syndrome coronavirus 2 (SARS-CoV-2) continues to surge. The emergent variants that can evade the immune response significantly affect the effectiveness of mainstream vaccines and diagnostic products based on the original spike protein. Therefore, it is essential to focus on the highly conserved nature of the nucleocapsid protein as a potential target in the field of vaccines and diagnostics. In this regard, our review initially discusses the structure, function, and mechanism of action of N protein. Based on this discussion, we summarize the relevant research on the in-depth development and application of diagnostic methods and vaccines based on N protein, such as serology and nucleic acid detection. Such valuable information can aid in designing more efficient diagnostic and vaccine tools that could help end the SARS-CoV-2 pandemic.

## 1. Introduction

The diagnosis and prevention of Severe acute respiratory syndrome coronavirus 2 (SARS-CoV-2) infection are crucial to control the spread of the virus and minimize mortality (Li et al., 2022; Zhang et al., 2023). SARS-CoV-2 is a highly contagious virus that mainly targets the respiratory and gastrointestinal systems, with aerosols and respiratory droplets being major modes of transmission (Harrison et al., 2020; Asselah et al., 2021; To et al., 2021). Detecting infected individuals early is paramount to effectively managing the pandemic. Given the significance of coronavirus spike protein (S) in SARS-CoV-2’s life cycle, which encodes four structural and seventeen non-structural proteins, it has received considerable attention (Ahammad and Lira, 2020; Brosseau et al., 2022). However, many other SARS-CoV-2 proteins play a similar noteworthy role in the virus’s lifecycle, though their structures and biophysical properties are largely unknown (Supekar et al., 2021; Zinzula et al., 2021). Of these, Nucleocapsid (N) protein is an attractive antiviral target.

Research indicates that patients infected with SARS-CoV-2 show a greater and earlier antibody response to the N protein than the surface spike (S) protein, despite the N protein being located inside the virus particle (Zeng et al., 2020). The N protein has been shown to possess non-specific nucleic acid binding capabilities, making it a potential diagnostic tool detection target (Li and Li, 2021). As for vaccine design, despite SARS-CoV-2 being prone to mutation, the high degree of conservation in the nucleocapsid protein gene sequence ensures a certain level of cross-protective efficacy, enabling the vaccine to protect against virus variations to some extent and mitigate the severity of illness (Premkumar et al., 2020; Shaw et al., 2021; Li et al., 2022; Rak et al., 2023). Moreover, the relatively simple structure of the nucleocapsid protein makes it easier for vaccine researchers to grasp as a target antigen and consequently develop highly specific and immunogenic vaccines (Du et al., 2009; Walls et al., 2020). Therefore, it is essential to summarize the relevant research on the nucleocapsid proteins of SARS-CoV-2.

This review introduces the life cycle of SARS-CoV-2, the structure and function of the N protein domain, and the mechanism of N protein action firstly. On this basis, it focuses on summarizing relevant research on the development and application of vaccines and diagnostic methods based on the N protein (including serological and nucleic acid detection). These valuable information will help in designing more efficient vaccines and diagnostic reagents, thereby promoting the end of the SARS-CoV-2 pandemic.

## 2. Structure and detection of SARS-CoV-2 N protein

### 2.1. The structure and biology of SARS-CoV-2 N protein

Presently, there are seven types of coronaviruses that infect humans worldwide. Since the isolation of Human Coronavirus (HCoV-229E) in 1965, our understanding of coronavirus has significantly improved. Coronaviruses have the ability to infect both animals and humans (Li and Li, 2021). The coronavirus particle has a diameter of 80–120 nm, a methylation cap structure positioned at the 5′ end of its genome, and a poly(a) tail located at the 3′ end. With an overall size between 27 and 32 KB, the genome is the largest RNA virus currently recognized (Kokic et al., 2021). Middle East respiratory syndrome (MERS), COVID-19, and severe acute respiratory syndrome (SARS) are all infectious diseases transmitted by the coronavirus and are prevalent worldwide (Kokic et al., 2021; Li and Li, 2021).

**FIGURE 1:**
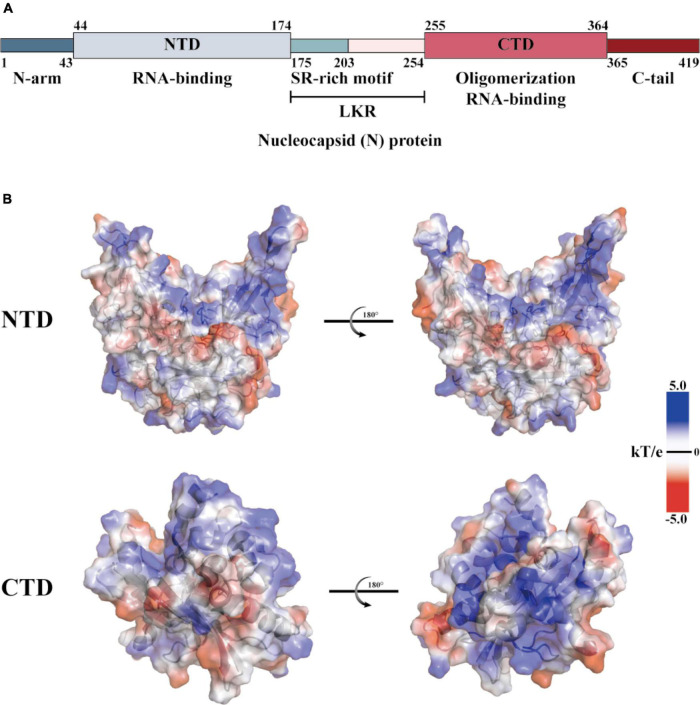
Structural overview of the SARS-CoV-2 N protein. **(A)** Schematic diagram of the modular structure of the N protein of SARS-CoV-2. The N-arm, the Ser/Arg (SR)-rich central LKR, and Ctail, and the N-terminal domain (NTD) and C-terminal domain (CTD)–the three intrinsically disordered regions–are depicted. **(B)** SARS-CoV-2 N-NTD (PDB ID: 7CDZ) and N-CTD (PDB ID: 7DE1) electrostatic surfaces (PDB ID 7CEO). Blue represents a positively charge potential, and red represents a negative one. PyMOL was utilized to prepare all the structural figures.

**FIGURE 2:**
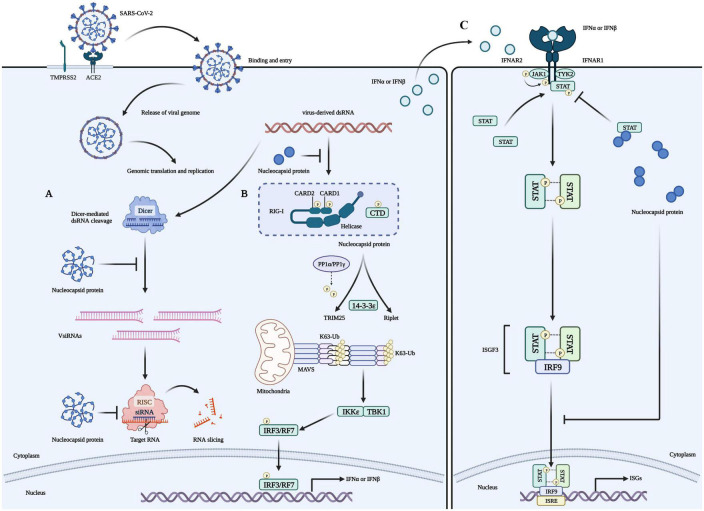
Role of the N protein in the innate immune processes of host cells. **(A)** The N protein is a crucial factor that inhibits RNA interference (RNAi) in host cells infected with RNA viruses. It exerts this inhibition through two mechanisms: Firstly, the N protein binds with double-strand RNA (dsRNA) to prevent its recognition and cleavage by Dicer, thus preventing RNAi at the initial stage. Secondly, the N protein can also inhibit RNA degradation caused by small interfering RNA (siRNA) during the effector stage of RNAi. These mechanisms contribute to the stable preservation of viral RNA and enable the replication and proliferation of viruses in host cells, ultimately causing severe damage to them. **(B)** Viruses use their N proteins to interact with RIG-I and suppress the innate immune response by inhibiting IFN-β production in host cells. This interaction is mediated through the helicase domain of DExD/H-box helicases. SARS-CoV-2’s N proteins inhibit IFN-β by targeting its initial activation step. **(C)** SARS-CoV-2’s N proteins can impede the host’s innate immune response by functioning as type I interferon (IFN) signaling antagonists, ultimately compromising the immune system’s ability to fight against the virus. Specifically, they inhibit the phosphorylation and nuclear translocation of the transcription factors STAT1 and STAT2. This results in the subsequent attenuation of ISGF3, the IFN-stimulated gene factor 3 transcription complex, and a reduction in the expression of genes that are activated by IFN. Consequently, the presence of N proteins in SARS-CoV-2 infected cells reduces the immune system’s activity, which may increase the severity of the disease in some people.

The coronaviridae family, which includes SARS-CoV-2, consists of non-segmented, single-stranded, positive-strand RNA viruses (Wang M. et al., 2020). This family encodes four structural proteins (S, E, M, and N), 16 non-structural proteins (Nsp1 to 16), and nine helper proteins (Badua et al., 2021; Dai and Gao, 2021). The N protein comprises intrinsically disordered regions (IDRs) and conserved structural parts, and its functional partitioning is based on sequence characteristics. The IDR includes three modules: the N-arm, the linker region (LKR), and the C-tail, while the conserved structural part is made up of the N-terminal domain (NTD) and the C-terminal domain (CTD) (Chang et al., 2006; Yao et al., 2020; Ma et al., 2021; Sarkar et al., 2022). The specific amino acid sequence of N and a schematic representation of the electrostatic surface of the NTD and CTD are illustrated in Figure 1. The N protein is the core component of the virus particles, which combines with viral genomic RNA and packages it into the ribonucleoprotein (RNP) complex (Ye et al., 2020). In addition to assembly, the N protein plays an important role in viral mRNA transcription and replication and participates in immune regulation (Bai et al., 2022). After SARS-CoV-2 infiltrates the host cell, the N protein separates from the virus’s positive-strand RNA genome, initiating a highly regulated gene replication and expression program (Baggen et al., 2021). The host cell has a natural antiviral immune defense mechanism known as RNA interference (RNAi), which degrades the virus genome to inhibit replication, but the N protein acts as a viral inhibitor of RNAi (Xin et al., 2017; Setten et al., 2019; Mu et al., 2020). The virus’s ORF1a and ORF1b are translated into polyproteins pp1a and pp1ab, respectively, from its (+) strand RNA genome. These polyproteins are cleaved by cysteine proteases nsp3 (papain-like protease, PL*^pro^*) and nsp5 (chymotrypsin-like protease, 3C protease 3CL*^pro^*, or major proteolytic enzyme, M*^pro^*) to produce non-structural proteins. NSP1 is one of the non-structural proteins generated and plays a role in inhibiting mRNA translation while speeding up mRNA decay in the host (Perlman and Netland, 2009; Li, 2016; Baggen et al., 2021; V’Kovski et al., 2021). While NSP2-16 and the N protein establish a viral replication-transcriptional complex and reshape the cell membrane to form replicating organelles connected to the endoplasmic reticulum (Perlman and Netland, 2009; Li, 2016; Ugurel et al., 2020; Baggen et al., 2021; Khan et al., 2021; V’Kovski et al., 2021). Viral RNAs are replicated in double-membrane vesicles, and new virions are formed by budding into the lumen at ERGIC (Baggen et al., 2021). The mechanism by which N protein interacts with the host is shown in Figure 2 The N protein regulates host cell cycle progression, host-pathogen interactions, and apoptosis, which plays a crucial role in the virus life cycle and the integration of viral RNA into offspring particles (de Haan et al., 1998; Surjit et al., 2005; McBride et al., 2014; Kwarteng et al., 2020). Therefore, the N protein is an essential component in the virus’s ability to infect host cells.

Antibodies against cytokine storms and SARS-CoV-2 have been the focus of intensive study at research institutions since the COVID-19 outbreak (Ragab et al., 2020; Kim et al., 2021). Their primary area of research interest has been the S protein, which acts as a receptor for the virus, and the virus can be prevented from entering cells by binding to neutralizing antibodies against the S protein or ACE2 protein (South et al., 2020; Verdecchia et al., 2020). The N protein is recognized for its strong immunogenicity and its ability to stimulate the immune system to produce protective responses. It is expressed at high levels during infection and is an ideal antigen for T-cell response, with the potential to trigger specific T-cell proliferation and cytotoxic activity in the context of a vaccine (Gao et al., 2003; Okada et al., 2005; Surjit and Lal, 2008; Supekar et al., 2021). Additionally, the N gene is highly conserved and stable, with 90% amino acid homology and minimal mutations over time (Drosten et al., 2003; Holmes and Enjuanes, 2003; Marra et al., 2003; Rota et al., 2003; Zhu et al., 2005; Grifoni et al., 2020). In the post-pandemic era, the N protein is not only a crucial target for fast and early diagnosis but also holds significant potential in the development of vaccines.

### 2.2. Infection and sample collection of SARS-CoV-2

SARS-CoV-2 can elicit an immune response in the body, often with immunoglobulin M (IgM) as the first line of protection (occurring within 3–5 days of infection). Immunoglobulins (IgG) often appear 1 week after infection, with high affinity and adaptive response, lasting for a long time, and can be used as a marker of previous infection. At present, the virus samples collected mainly from the upper respiratory tract, lower respiratory tract and blood. Sometimes, digestive tract samples are also used. Upper airway specimens included nasopharyngeal swabs (NPS), oropharynx swabs (OPS), tongue swabs (LS) and mouthwash samples, while lower airway specimens included sputum, tracheal aspiration (TA) and Bronchoalveolar lavage fluid (Balf) Depending on the kit, the blood sample can be whole blood or serum, and the digestive tract sample usually includes an anal swab (Wang et al., 2021).

### 2.3. The influence of sample collection on detection results in practical application

The site and method of collection will affect the viral load collected. NPS are generally considered to be the site of collection with the highest viral load (Lee E. et al., 2021). However, studies have also shown that saliva samples are more sensitive than NPS in diagnosing asymptomatic or mild viral infections, particularly in detecting such infections in children and adults (Wang et al., 2021).

The choice of collection method and the absence of lysis also have a significant influence on the detection results. Currently, the World Health Organization recommends that collected swabs should be placed in collection tubes containing viral transport media or sterile saline solution. However, some researchers believe that substituting lysis buffer instead of virus storage solution may improve the safety and sensitivity of detection (Erster et al., 2021). Additionally, researchers have developed a technique called precipitation-enhanced analyte recovery of Lysate Solutions, which enables rapid separation of nucleic acids and proteins from a variety of sources in a sample. Furthermore, it has the advantages of high sensitivity, affordability, and rapidity, and it can be used in point-of-care testing (POCT) (Ponce-Rojas et al., 2021). Serological testing kits typically only require storage at room temperature, making them suitable for POCT. On the other hand, nucleic acid detection kits necessitate cold chain transportation, leading to increased costs. As a result, serological testing kits are the preferable choice for POCT (Cheng et al., 2023).

## 3. Application of N protein in the detection of SARS-CoV-2

The routine detection of SARS-CoV-2 mainly includes serological detection and nucleic acid detection. In PCR-positive patients, the N protein was detected more frequently than the S protein (Okba et al., 2020). The SARS-CoV-2 nucleocapsid protein is abundant in virions and in infected individuals. The N protein gene is more conserved and stable than the S protein gene, with fewer mutations over time. Therefore, almost all PCR detection reagents and most serological detection kits use the N protein as the detection target (Bouhaddou et al., 2020; Grifoni et al., 2020). Here, we provide a comprehensive review of the practical techniques to detect SARS-CoV-2 and list the approved relevant assay kit information for each technique. The specific evaluation results are shown in Table 1.

**TABLE 1:** Evaluation of the advantages/disadvantages of SARS-CoV-2 detection technology.

Methods	Assay name	Targeted genes	Sample type	Minimum detection limit	TAT/ test	Advantages	Disadvantages	References
**Nucleic acid-based molecular biology diagnostics**								
RT-PCR	Cobas^®^ SARS-CoV-2 Test (Roche)	ORF-1a, E	Nasopharyngeal and oropharyngeal swabs	Target 1: 25 copies/mL (95% CI: 17wabsiopies/mL); Target 2: 32 copies/ml (95% CI: 21–73 copies/mL)	3–4 h	High specificity and sensitivity	High rate of false negatives, and has experimental operation and cost requirements	Staff, 2016
RT-PCR	ID NOW COVID-19 assay (Abbott)	RdRp	Nasal, Throat, and Nasopharyngeal swabs	NA	≤ 13 min			Farfour et al., 2021
RT-LAMP	Biofire Filmarray RP-2.1 (bioMerieux)	RdRp, N, E	Nasopharyngeal swab in transport media or saline	NA	∼45 min	No need to raise or lower temperature; short reaction time; simple procedure	Non-specific amplification is difficult to identify	Martinez et al., 2016
CRISPR-Cas system	CRISPR-Cas12-based assay (Cepheid)	N, E	Respiratory swabs	NA	≤ 40 min	Suitable for point-of-care testing (POCT)	Possible care target” phenomenon	Broughton et al., 2020
**Serological diagnosis based on antigen-antibody**								
FIA	Sofia 2 Flu + SARS Antigen FIA (Quidel Corporation)	N	Nasal swab, Nasopharyngeal swab	91.7 TCID50/ml	15–20 min	High sensitivity, simple reagents	Window period, easy to miss diagnosis, cross reaction	Brihn et al., 2021
MESIA	Sampinute COVID-19 Antigen MIA (Celltrion USA, Inc.)	S	Nasopharyngeal swab	1.2 × 10^2^ TCID50/ml	15–20 min	Magnetic control, high sensitivity	target is susceptible to virus mutation.	Mahmoudinobar et al., 2021
LF-CGIA	BinaxNOW COVID-19 Ag Card Home Test (Abbott Diagnostic Scarborough Inc.)	N	Nasal swab	140.6 TCID50/ml	15–20 min	Positive results are very accurate, easy to operate	Negative does not rule out infection	Okoye et al., 2021
CDI	BD Veritor System for Rapid Detection of SARS-CoV-2 (Becton, Dickinson and Company (BD))	N	Nasal swab	1.4 × 10^2^ TCID50/ml	15–20 min			Van der Moeren et al., 2020
**Diagnostic medical imaging**								
CT	/	/	/	/	About 1 h	More accurate in determining disease status	Cannot be distinguished from other viral pneumonia	
Artificial intelligence: CT combined with algorithm-based deep learning	/	/	/	/	About 1 h	Diagnostic capability based on continuous optimization of algorithms	AI recognition models need to pass a certain time in training, and the technical requirements are high	
Viral culture	/	/	/	/		Strains can be isolated for other experiments; High accuracy of culture results	Very time-consuming; labor-intensive; requires high professional knowledge of operators; requires high biological safety	

### 3.1. Application of the N protein in serological detection

**FIGURE 3:**
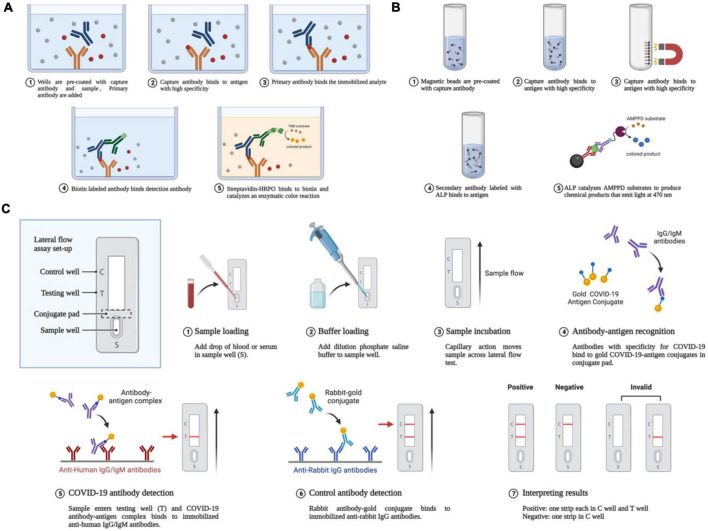
Antigen-antibody-based serological detection of SARS-COV-2. **(A)** Traditional ELISA method based on a double antibody sandwich assay. **(B)** Magnetic beads adsorption of antigens and antibodies. **(C)** Using a lateral flow assay or double antibody sandwich, the quick quantum dot and colloidal gold immunodiagnostic approach for SARS-CoV-2 antibody is based on high specificity for the recombinant protein and quantum dot/colloidal gold immunofluorescence probes.

Recently developed cutting-edge serological tests are both sensitive and specific (Lu et al., 2020). In patients with negative RT-PCR results, but with suspected COVID-19, serological testing for antibodies against the pathogen and specific antigens can help further determine the existence of infection. In addition, compared with nucleic acid tests, serological tests tend to be more practical in terms of cost, use, and speed. To date, various techniques have become available for the fast detection of SARS-CoV-2-specific antigens and antibodies. Among them are enzyme-linked immunosorbent assay (ELISA), chemiluminescent immunoassay (CLIA), lateral flow immunoassay (LFIA), the luciferase immunoprecipitation system assay (LIPS), and the rapid antigen detection (RAD) assay (Rastawicki and Rokosz-Chudziak, 2020). Using a RAD kit, patients can even test themselves at home. Respiratory secretions can be tested for the presence of antigens produced by SARS-CoV-2 proteins using these methods (Gourgeon et al., 2022). The steps for the various types of serological detection methods are shown in Figure 3.

#### 3.1.1. RAD method

The NP antigen is considered one of the most reliable early diagnostic markers for SARS-CoV-2, as it can be detected up to 1 day before the onset of clinical symptoms (Che et al., 2004). RAD methods have been developed to detect the SARS-CoV-2 N protein in respiratory samples, including nasopharyngeal swab samples. Diao et al. (2021) developed a rapid and convenient method for detecting SARS-CoV-2 NP antigen using fluorescence immunochromatographic (FIC) assay, which exhibited high sensitivity (75.6%, 95% CI: 69.0–81.3) and specificity (100%, 95% CI: 91.1–100) among participants with a Ct value < 40. Various research groups have investigated the sensitivity of RAD tests to detect SARS-CoV-2 N antigen, reporting a range of 13% to 62%. A meta-analysis conducted by Fragkou et al. (2023) revealed that when antigen-based RAD tests were used as a screening tool in the general population, sensitivity decreased to 49.3% (95% CI: 39.7–59.1%). Similarly, sensitivity decreased to 46.2% (95% CI: 36–56.6%) when the test was performed more than 7 days after symptom onset. Another meta-analysis by the Veroniki et al. (2023) team, involving 36 rapid antigen tests with 104,961 participants, emphasized that the method of sample collection could impact test sensitivity. Nasal or combined samples (e.g., combinations of nose, throat, mouth, or saliva samples) yielded higher sensitivity, while nasopharyngeal samples and samples from asymptomatic individuals during testing resulted in lower sensitivity.

#### 3.1.2. ELISA method

When detecting anti-SARS-CoV-2 antibodies, ELISA is the go-to test (Zhang et al., 2020). Using ELISA, Ogata et al. (2020) reported that 64.1% of plasma from COVID-19-positive patients contained the SARS-CoV-2 N antigen. A German study examined respiratory swabs from 107 PCR-positive and 303 PCR-negative individuals for SARS-CoV2 N antigen. Clinical isolates B.1.117, variant of concern (VOC) Alpha (B.1.1.7), or VOC Beta (B.1.351) were also analyzed. Specificity was 99.7%, whereas sensitivity was 17.8 percent. ELISA also detected the alpha and beta VOCs N antigens of SARS-CoV2 (Osterman et al., 2021).

#### 3.1.3. CLIA method

The N protein of SARS-CoV-2 can be qualitatively or quantitatively detected in samples using CLIA, an automated high-throughput approach. An anti-N antibody is first used to bind the sample’s N protein antigen to magnetic particles. Prince-Guerra et al. (2021) evaluated the clinical utility of CLIA testing. The study found that almost all nasopharyngeal swabs (92.6%) with a Ct value of 35 tested positive using CLIA. The assay has a sensitivity of 87.9% and a specificity of 95.6%.

### 3.2. Nucleic acid detection based on the N gene

**FIGURE 4:**
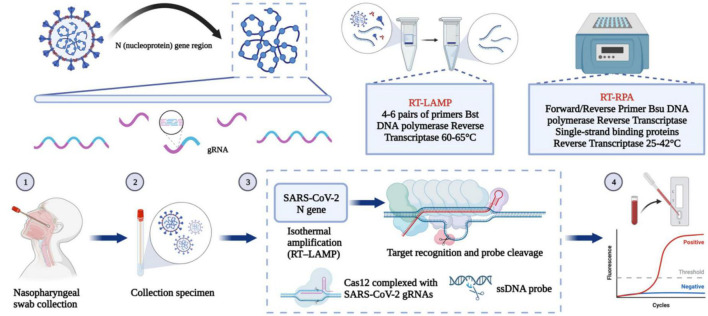
Nucleic acid-based detection of SARS-CoV-2. CRISPR/Cas system: Purified RNA can be amplified in an isothermal instrument using either reverse transcription recombinant polymerase amplification (RT-RPA) or reverse transcription loop-mediated isothermal amplification (RT-LAMP). The amplified product can be reported using either the chromogenic substances in the amplification system or the CRISPR/Cas system for additional specific cleavage of nucleic acids and determination of virus infection. After the guide RNA binds to the CRISPR-associated Cas protein, the resulting complex can specifically cleave the viral nucleic acid sequence. The result can be reported by fluorescence quenching molecules in the reaction, by reporting the fluorescence signal, or by the side stream chromatography color development strip of the cleaved nucleic acid fragment.

Reverse transcription PCR (RT-PCR) is a common nucleic acid detection method. This method uses different gene fragments of SARS-CoV-2 to design corresponding primers and probes to identify viral genetic material. There are few mutations in the N gene; therefore, PCR kits designed based on the N gene are suitable to judge whether a sample comes from a person infected with SARS-CoV-2 (Zhou et al., 2022). In addition to PCR technology, this section will also introduce a variety of nucleic acid detection methods based on the N gene. The steps for several important methodological approaches are shown in Figure 4. At the same time, more and more companies have developed updated nucleic acid-based SARS-CoV-2 detection kits (Table 2).

**TABLE 2:** Commercial rRT-PCR test kits for diagnosing SARS-CoV-2 infection.

Assay name	Company (country)	Targeted genes	Sample type	Minimum detection limit	TAT/test	Approval	References
2019-nCoV Nucleic acid detection kit (Fluorescence RT-PCR)	Sansure Biotech Inc. (China)	ORF-1ab, N	Pharynx swab, bronchoalveolar lavage fluid	200 copies/mL	3–4 h	China (NMPA)	Sansure Biotech Inc, 2022
SARS-CoV-2 Nucleic Acid Detection Kit (Fluorescence RT-PCR)	ComWin Biotech Co., Ltd (China)	ORF-1ab, N, actin	Throat swab, nasal swab, nasopharyngeal extract, deep cough sputum	1 × 10^3^ copies/mL	3–4 h	China (NMPA)	Cwbio, 2020
2019-nCoV Nucleic acid detection kit (Fluorescence RT-PCR)	Shanghai GeneoDx Biotech Co., Ltd (China)	ORF-1ab, N	Nasopharyngeal swabs, sputum, bronchoalveolar lavage fluid	500 copies/mL	∼30 min	China (NMPA)	Wang X. et al., 2020
Cobas^®^ SARS-CoV-2 Test	Roche (Switzerland)	ORF-1a, E	Nasopharyngeal and oropharyngeal swabs	Target 1: 25 copies/mL (95% CI: 17wabslveolar la Target 2: 32 copies/ml (95% CI: 21–73 copies/mL)	3–4 h	US FDA- EUA CE-IVD	Staff, 2016
ID NOW COVID-19 assay	Abbott (USA)	RdRp	Nasal, Throat, and Nasopharyngeal swabs	NA	≤ 13 min	US FDA- EUA	Farfour et al., 2021
CRISPR-Cas12-based assay	Cepheid (USA)	N, E	Respiratory swabs	NA	≤ 40 min	NA	Broughton et al., 2020
Biofire Filmarray RP-2.1	bioMerieux (France)	RdRp, N, E	Nasopharyngeal swab in transport media or saline	NA	∼45 min	No	BioFire, 2023

NMPA, National Medical Products Administration; CE-IVD, Conformite Europeenne *in vitro* diagnostic device; EUA, Emergency Use Authorization; US FDA, Food and Drug Administration of the United States; NA, not available.

#### 3.2.1. RT-PCR designs based on site-directed mutagenesis

Considering the continuous evolution of the virus, RT-PCR technology designed for mutation sites is crucial to detect mutant strains. For example, Vega-Magaña et al. (2021) designed three specific primers and probes that could detect the 69–70Del and K417N mutations with high prevalence in the N gene. In addition, another study used similar method to design a PCR kit for the rapid detection of key mutations of the latest Omicron variant, such that it can be distinguished from other variants (Zelyas et al., 2021).

However, the assay still possesses certain limitations, including inadequate viral load in samples, disparities in kit quality, and laboratory errors associated with sample collection or test execution. Moreover, individuals who are recuperating from SARS-COV-2 might also exhibit positive RT-PCR results, which suggests the existence of potential false positives.

#### 3.2.2. Reverse transcription Loop-mediated isothermal amplification (RT-LAMP)

Loop-mediated isothermal amplification has also been developed as a fast, robust, and cheap technology, and is now considered a reliable alternative to traditional RT-PCR diagnosis (Kashir and Yaqinuddin, 2020). RT-LAMP does not need expensive thermal cycling equipment and is a portable and rapid detection method. In addition, the technology is also highly specific because it uses about 6–8 specific primer sequences to identify eight different regions of the target (Hanson et al., 2020). The sensitivity and specificity of RT-LAMP in detecting mutants are worthy of attention. For example, Almeida et al. (2022) successfully verified that RT-LAMP targeting the N and E genes could effectively detect the Omicron SARS-CoV-2 variant and its subline. In addition, another study tested 267 sequenced RNA genomes from different Omicron sublines using RT-LAMP technology. The results showed that the detection sensitivity, specificity, and accuracy of RT-LAMP for the BA.1 and BA.2 lineages and their derived mutants were close to 100%. These results suggested that RT-LAMP might become an alternative method to help monitor mutations, especially in countries with scarce resources.

#### 3.2.3. Nucleic acid detection method based on CRISPR-Cas

CRISPR/CAS technology also plays an important role in the specific detection of mutants. Nguyen et al. (2022) detected and identified SARS-CoV-2 VOCs (including Alpha, Beta, Gamma, Delta, and Omicron) based on CRISPR-Cas12b, and verified them in 208 clinical samples. The sensitivity, specificity and accuracy of CRISPR-SPADE were 92.8, 99.4, and 96.7% in 10–30 min. For samples with a high viral load (Ct ≤ 30), 100% accuracy and sensitivity were obtained. In addition, Liang et al. (2022) also developed a detection method based on CRISPR-Casl2a. They designed allele-specific CRISPR RNA to target specific mutation sites of the Omicron variants, to specifically detect Omicron mutants. In short, the introduction of CRISPR/CAS technology has overcome the adverse effects of cross reaction and virus mutation on detection performance to a certain extent, and thus has a good application prospect.

The development of LAMP and CRISPR/Cas-based POC detection methods allows for the delivery of results within an hour, which is advantageous in terms of time efficiency. The introduction of CRISPR/CAS technology partially mitigates the negative impact of cross-reactions and virus mutations on detection performance. In comparison to the time-consuming and costly whole genome sequencing of mutants, the high-performance LAMP and CRISPR/CAS kits exhibit promising potential in the current efforts to promptly diagnose and track Omicron mutants. Consequently, the implementation of these techniques enhances the classification of diverse infectious mutants, allowing for the strategic allocation of medical resources and adjustments to epidemic prevention policies. While serological-based rapid antibody detection enables large-scale immune screening, it is still limited in terms of its time delay and inability to conclusively prove the presence of the virus.

## 4. Challenge of N protein in the detection of SARS-CoV-2

**FIGURE 5:**
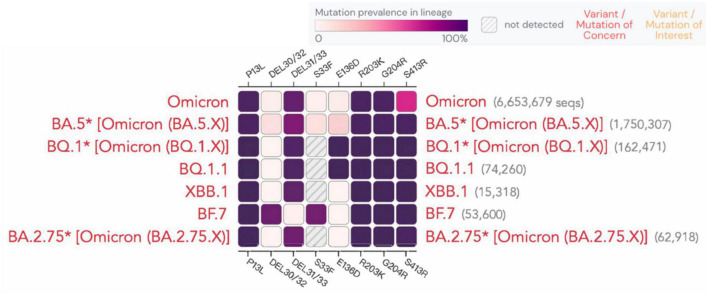
Mutation prevalence across lineages. The Outbreak platform provides N protein mutation prevalence across the major subtypes of Omicron strains across lineages. (Mutations with > 75% prevalence in at least one lineage) (https://outbreak.info). The figure describes in detail the mutation prevalence in the Omicron lineage. Blank indicates that the mutation has not been detected. White to purple indicates the prevalence of the mutation in all sequences.

According to a recent study (Meiners et al., 2022), the sensitivity of rapid antigen tests decreased over time, dropping from 80 to 67%. Although the N protein is relatively conserved, mutations at certain sites are still observed, as shown for different variants in Figure 5. A study demonstrated that the N-terminal mutation R203K/G204R in the SARS-CoV-2 nucleocapsid protein causes protein aggregation, enhances RNA binding ability, and leads to the overexpression of host cell interferon-related genes. This mutation has been linked to an elevated viral load in patients with COVID-19. R203K/G204R mutations enhance the virus’s sensitivity to neutralizing antibodies, which may be strengthened by immune resistance mutations like N501Y and E484K (Mourier et al., 2022). Whether and how these sites affect antigen or nucleic acid detection is still being studied. Below, we present the latest advancements on the detection of the impact of new variants.

### 4.1. Reduced sensitivity to novel variants in most RATs when Ct values are used as the comparison

Concerns are raised regarding the performance, including sensitivity, of various kits as each variant emerges. Many nucleic acid or serum detection kits were developed using the original Wuhan-Hu-1 strain, making it important to periodically re-evaluate them to ensure that their performance meets the standard (Frank et al., 2022). Most detection kits used in recent studies demonstrated decreased sensitivity to new variants, particularly at low viral loads (Ct ≥ 25) (Table 3). The data indicate that many assay kits used in studies conducted by the Osterman A team showed decreased sensitivity toward the new variants at the same Ct values. For instance, the Glallergen assay kit exhibited a 28.9% decrease in sensitivity toward variant BA.1, while the FUJIFILM, CLINITEST, and Biosynex assay kits exhibited decreases of 53.6, 50, and 39.4%, respectively (Osterman et al., 2022, 2023). In contrast, no sensitivity decreases toward the new variants were observed in the assay kits tested (AAZ, AMP, Biospeedia, Siemens) in the study conducted by the Gourgeon et al. (2022) A team. Rao et al.’s (2023) research showed that most commercially available rapid antigen tests (RATs) had similar sensitivity in detecting the Omicron and Delta variants when antigen concentration was used as a basis for comparison. However, when sensitivity was compared using Ct values, most RATs were less sensitive to Omicron than Delta. This is consistent with the laboratory research results presented in Table 3, which used different Ct values to compare sample sensitivity. However, it is essential to note that RAT testing detects antigens, not RNA. Therefore, if different variants have varying antigen concentrations, even with the same RNA copy number, the results of experiments comparing sensitivity using Ct values may be biased. In fact, Rao et al.’s (2023) team found that the Omicron samples had a lower antigen-to-RNA ratio than the Delta samples. Differences in RNA and antigen concentrations could be influenced by factors such as differences in vaccine booster timing, variant-specific differences in the viral lifecycle, and the time interval between infection and sample collection for testing.

**TABLE 3:** Comparison of performance of COVID-19 N antigen detection kits in different variants.

Study name (Manu-facturer)	Test name	Detec -ted anti -gen	Overall sensitivity				Sensitivity (Ct ≤ 25)				Sensitivity (Ct ≥ 25)				Specifi- city	Refe- rences
			**Non-** **Delta/** **non-** **Omicron**	**Delta**	**Omic-** **ron** **BA.1**	**Omic-** **ron** **BA.2**	**Non-** **Delta/** **non-** **Omicron**	**Delta**	**Omic-** **ron** **BA.1**	**Omic-** **ron** **BA.2**	**Non-** **Delta/** **non-** **Omicron**	**Delta**	**Omic-** **ron** **BA.1**	**Omic-** **ron** **BA.2**		
Clinitest (Siemens Healthineers)	The Clinitest^®^ Rapid COVIDNA19 Antigen test	N, S1, S1 NARBD, S2	NA	72.9%	69.6%	NA	NA	95.6%	94.1%	NA	NA	32%	0%	NA	NA	Bayart et al., 2022
NewNAGene (NewNAgene Bio engineering)	The NewNAgene COVIDNA19 Antigen Detection Kit	N	NA	75.7%	73.9%	NA	NA	95.6%	97.1%	NA	NA	40%	7.7%	NA	NA	
Boson (Xiamen Boson Biotech Co.)	The Boson Rapid SARSNA CoVNA2 Antigen Test Card	N	NA	77.1%	78.3%	NA	NA	97.8%	97.1%	NA	NA	40%	23.1%	NA	NA	
Flowflex (Acon Laboratories)	The Flowflex COVIDNA19 Antigen Home Test	N	NA	70%	67.4%	NA	NA	97.8%	91.2%	NA	NA	20%	0%	NA	NA	
Sejoy (Hangzhou Sejoy Electronics and Instrument Co.)	The Sejoy SARSNA CoVNA2 Antigen Rapid Test Cassette	N	NA	74.3%	73.9%	NA	NA	95.6%	97.1%	NA	NA	36%	7.7%	NA	NA	
Roche (Roche Diagnostics)	The Roche SARSNA CoVNA2 Rapid Antigen Test Nasal	N	NA	92.9%	78.3%	NA	NA	100%	100%	NA	NA	80%	23.1%	NA	NA	
Clongene (Hangzhou Clongene Biotech Co)	LungeneNA COVIDNA19 Antigen Rapid Test Cassette	N	50%	NA	67.1%	56.5%	94.4%	NA	91.1%	76.1%	34.8%	NA	21.7%	18.2%	98.08%	Osterman et al., 2023
nal von minden (nal von minden GmbH)	Nadal COVIDNA19 Ag Test	N	36%	NA	58.6%	58.6%	83.3%	NA	86.7%	76.6%	13%	NA	8.7%	22.7%	98.08%	
Glallergen (Glallergen Co)	Novel Corona Virus (2019NAnCoV) Antigen Test Kit	N	92%	NA	48.6%	50%	100%	NA	71.1%	68.1%	100%	NA	8.7%	13.6%	96.15%	
Saier (Suzhou Soochow University Saier Immuno Biotech Co.)	InstantSure COVIDNA19 Ag CARD	N	96%	NA	55.7%	55.7%	100%	NA	80%	74.5%	100%	NA	13%	18.2%	98.08	
Egens (Nantong Egens Bio-technology Co)	EGENS SarsNA CoVNA2 Antigen Rapid Test	SARSNA CoVNA2 antigen	90%	NA	37.7%	35.7%	100%	NA	52.3%	44.7%	100%	NA	13%	18.2	98.08%	
FUJIFILM (Fujifilm Cooperation)	FUJIFILM COVIDNA19 Ag Test	N	38%	34.9%	22.2%	NA	85%	50%	31.4%	NA	10%	5.6%	0%	NA	100%	Osterman et al., 2022
Hotgen (Beijing Hotgen Biotech Co., Ltd.)	Novel Coronavirus 2019NAnCoV Antigen Test (Colloidal gold)	N	56%	56.2%	35.6%	NA	100%	74.4%	50%	NA	47.8%	22.2%	0%	NA	100%	
NanoRepro (NanoRepro AG)	NanoRepro SARSNA CoVNA2 Antigen Schnelltest (Viromed)	N	NA	53.9%	31.7%	NA	94.1%	72.7%	44.4%	NA	NA	16.7%	0%	NA	100%	
CLINITEST (Healgen Scientific LLC)	CLINITEST Rapid COVIDNA19 Antigen Test	N	76%	55.4%	35.6%	NA	100%	72.7%	50%	NA	87%	22.2%	0%	NA	100%	
Lyher (Hangzhou Laihe Biotech Co., Ltd.)	Lyher Novel Coronavirus (COVIDNA19) Antigen Test Kit (Colloidal Gold)	N	42%	58.5%	47.5%	NA	94.4%	77.3%	63.9%	NA	17.4%	22.2%	8.3%	NA	100%	
Biosynex (Biosynex Swiss SA)	COVIDNA19 Ag BSS selfNAtest	N	74%	55.4%	43%	NA	100%	75%	60.6%	NA	78.3%	16.7%	0%	NA	100%	
Boson (Xiamen Boson Biotech Co., Ltd.)	rapid SARSNA CoVNA2 Antigen Test Card	N	54%	49.2%	29.7%	NA	100%	72.1%	41.7%	NA	43.5%	0%	0%	NA	100%	
MP (MP Biomedicals Germany GmbH)	rapid SARSNA CoVNA2 Antigen Test Card	NA	54%	58.5%	51.5%	NA	100%	75%	70.8%	NA	43.5%	27.8%	4.2%	NA	100%	
Medicovid NAAG (Xiamen Boson Biotech Co., Ltd.)	Medicovid NAAG SARSNA CoVNA2 Antigen Rapid Test CardNAnasal	NA	NA	5 5.4%	57.4%	NA	NA	75%	77.8%	NA	NA	16.7%	8.3%	NA	100%	
Abbott N (Abbott Laboratories)	Abbott SARSNA CoVNA2 IgG assay	N	NA	NA	NA	NA	NA	NA	91.3%	NA	NA	NA	NA	NA	NA	Migueres et al., 2023
InBios (InBios International Inc)	SCoVNA2 Ag Detect Rapid SelfNATest	N	81.2%	90.7%	83.6%	NA	NA	NA	NA	NA	NA	NA	NA	NA	99.8%	Bekliz et al., 2022
Panbio (Abbott)	Panbio COVIDNA19 Ag Rapid test device	NA	NA	67.7%	36.1%	NA	NA	NA	NA	NA	NA	NA	NA	NA	NA	Bekliz et al., 2022
SD Biosensor (Roche)	Standard Q COVIDNA19 Ag	N	NA	52.9%	22.2%	NA	NA	NA	NA	NA	NA	NA	NA	NA	NA	
Sure Status (Premier Medical Corporation)	Sure Status	N	NA	52.9%	27.8%	NA	NA	NA	NA	NA	NA	NA	NA	NA	NA	
Wondfo (Wondfo)	2019NAnCoV Antigen test	N	NA	76.5%	75%	NA	NA	NA	NA	NA	NA	NA	NA	NA	NA	
Tigsun (Beijng Tigsun Diagnostics Co. Ltd)		N	NA	52.9%	47.2%	NA	NA	NA	NA	NA	NA	NA	NA	NA	NA	
CTK biotech (Onsite)	Onsite COVIDNA19 Ag Rapid Test	N	NA	64.7%	47.2%	NA	NA	NA	NA	NA	NA	NA	NA	NA	NA	
Flowflex (ACON biotech)		N	NA	91.2%	88.9%	NA	NA	NA	NA	NA	NA	NA	NA	NA	NA	
Bionote (Bionote)	NowCheck CovidNA19 Ag test	N	NA	NA	NA	NA	NA	NA	NA	NA	NA	NA	NA	NA	NA	
BinaxNOW™ (Abbott)	COVIDNA19 Ag AtNAHome Card	N	NA	41%	33%	NA	NA	41%	33%	NA	NA	12%	0%	NA	NA	Kanjilal et al., 2022
AAZ (AAZ)	COVID-NAVIRO antigen rapid test	N	92.3%	81.5%	89.5%	NA	95.8%	93.6%	100%	NA	50%	0%	69.2%	NA	100%	Gourgeon et al., 2022
AMP (AMP Diagnostics)	AMP rapid test SARSNA CoVNA2 Ag	N	94.2%	92.6%	92.1%	NA	95.8%	100%	100%	NA	75%	42.9%	76.9%	NA	100%	
Novel (Medakit)	Novel coronavirus (COVIDNA19) antigen test kit	N	90.0%	87.0%	89.5%	NA	95.7%	97.9%	100%	NA	25%	14.3%	69.2%	NA	100%	
Biospeedia (Biospeedia)	BSDNA0500 333NA25NA COVID19 speed antigen test	N	92.0%	88.9%	89.5%	NA	95.7%	100%	100%	NA	50%	14.3%	69.2%	NA	100%	
Siemens (Siemens Healthcare)	Test antigénique rapide Clinitest COVIDNA19	N	92.3%	85.2%	86.8%	NA	95.8%%	95.7%	100%	NA	50%	14.3%	61.5%	NA	100%	

Ct, Cycle threshold; Non-Delta/non-Omicron, The variants tested were neither Delta nor Omicron variants; NA, not available.

Most testing kits use the relatively conservative Nucleocapsid gene/protein as a detection target. However, the effectiveness of these kits has declined to varying degrees with the emergence of new variants. Repeatedly replacing the detection target for different variants can be both time-consuming and expensive. As a result, companies producing testing kits with greater declines in effectiveness should optimize the epitopes targeted by their products in a more cost-effective manner. Furthermore, test kit manufacturers should evaluate the ability of their products to detect new variants and publicly disclose relevant data. This would enable individuals to determine whether they have received a correct diagnosis.

### 4.2. Variations in the N gene can result in false negative results in nucleic acid testing

The continuous evolution of the virus could result in a decrease in the efficiency of RT-PCR detection. In emerging variants of concern (VOCs), the binding region for primers or probes may undergo high-frequency nucleotide changes, which could reduce the sensitivity of SARS-CoV-2 detection technology (Sun et al., 2022). Some primers and probes have demonstrated nucleotide mismatches. Currently, most of the newly developed, specific RT-PCR tests for VOCs available in the market target the mutation sites on the S protein gene (Lu et al., 2021; Chung et al., 2022). However, the impact of N gene mutations on the diagnostic performance of SARS-CoV-2 is scarcely investigated.

Alkhatib et al. (2022) showed for the first time that the Seegene Allplex SARS-CoV-2 assay failed to detect the target N gene target when using a regular RT-PCR kit. The deletion of G214-G215 on the N gene of this variant resulted in negative repeated detection results from nasopharyngeal swab samples. Furthermore, Chen et al. (2022) assessed the influence of numerous significant SARS-CoV-2 variations (including Omicron) on the analytical sensitivity of five commercial SARS-CoV-2 PCR detection methods. The results showed that the alpha and Omicron mutants had little effect on the detection performance. However, there are still some differences in specificity and sensitivity between different PCR kits, which depend on their target, primer design, and other factors (Kaden, 2020; Wang R. et al., 2020; Ziegler et al., 2020). Interestingly, many other studies and case reports have shown that the existing detection efficiency might be affected by N gene mutation. In the case of the Omicron mutant, several mutations were seen in various clusters of targets for N gene primers and probes in Japan, Thailand, and China (Kaden, 2020). All these results indicated that the N gene might not be the best stability index for RT-PCR kits.

Although PCR technology is the “gold standard” approach for viral identification, it does have some major drawbacks. For example, the varied viral load of various samples is a major reason for false negative RT-PCR results, which can have a significant impact on the prevention of virus transmission and epidemics. Furthermore, SARS CoV-2 rehabilitation patients might test positive for virus carriage using RT-PCR. In addition, many RT-PCR test kits call for expert-level biosafety level 2 laboratories and lengthy research and development cycles. This RT-PCR method is too complicated and time-consuming for easy, on-site diagnosis and screening of patients. Therefore, to stop the spread of the virus and guarantee timely treatment, we require detection equipment that can quickly identify many infected and asymptomatic carriers.

### 4.3. Emerging variants have limited impact on SARS-CoV-2 detection

The Filipp Frank team utilized a deep mutational scanning approach to measure the effect of all possible mutations in the Nucleocapsid protein on antibody binding in a single experiment, which generates a complete escape mutational profile for each antibody. These profiles help to identify distinct regions of high and low escape scores, indicating the epitopes and vulnerabilities of diagnostic antibodies to mutations within and outside of the epitope. Most variation did not impact antibody binding, but a small subset clustered at specific positions that considerably reduced binding. Frank et al.’s (2022) team assessed the performance of 17 diagnostic antibodies authorized for emergency use by the US Food and Drug Administration in SARS-CoV-2 rapid antigen tests and found no vulnerabilities for the detection of mutations found in variants of concern. However, the DMS library used by the team only included single mutations, and therefore, cannot accurately predict the effects of multiple mutations with synergistic effects. Furthermore, the presence of mutated spike antigens might cause conformational changes, leading to inconsistencies in assay performance for detecting SARS-CoV-2 variants in patient samples compared to recombinant antigens. Thus, further studies are necessary to evaluate the diagnostic assay’s effectiveness for detecting SARS-CoV-2 variants, particularly in patient samples (Frank et al., 2022; Tieman et al., 2022; Wertenauer et al., 2022).

### 4.4. Limitations of nucleocapsid proteins in strain detection

The N protein’s role in strain identification through sequencing is limited due to its relative conservation. Currently, the identification of new variants depends on next-generation sequencing (NGS) after a positive diagnosis. This technology enables the identification of nucleic acid sequences without prior knowledge of the specific mutations. Sequencing technology has contributed to the identification of multiple variants of concern (VOCs) (Chiara et al., 2021). During the initial phase of the pandemic, there were relatively few cases of SARS-CoV-2 infection, and numerous Centers for Disease Control and Prevention (CDC) were able to promptly monitor epidemic strains and detect newly emerging variants within a short timeframe. However, with countries gradually adjusting their epidemic prevention policies and experiencing a significant increase in infection numbers, local CDC sequencing and VOC tracking have faced considerable challenges. Rapid, sensitive, and user-friendly diagnostic tests are urgently required to detect SARS-CoV-2 and address the ongoing pandemic.

Antigen tests for detecting SARS-CoV-2 have emerged as a promising rapid diagnostic method for COVID-19. However, they are unable to differentiate the variants of concern (VOCs) of interest. Heggestad et al.’s (2023) team developed a rapid point-of-care test called CoVariant-SPOT, which utilizes a panel of antibodies tolerant or intolerant to spike protein mutations. This test identifies the likely SARS-CoV-2 strain concurrently with COVID-19 diagnosis by targeting the nucleocapsid protein. By examining the fluorescence intensity from various anti-S antibodies, it cleverly exploits the immune escape of different variant S proteins and successfully distinguishes between the Delta and Omicron VOCs. Although N protein testing cannot reveal the corresponding variants, it can serve as a positive indicator for strain identification. Strain identification not only facilitates personalized treatment but also significantly reduces the difficulty for researchers in screening the desired variant samples.

## 5. Nucleocapsid-based SARS-CoV-2 vaccines

The evolving challenge of VOCs and the limited persistence of immunity induced by first-generation vaccines necessitate the development of new COVID-19 vaccines urgently. Upgrading the S protein to handle VOCs is a complex and costly process due to its variability. Both T-cell immunity and neutralizing antibodies have been identified as key components in defending against both the original and VOC strains. In immunology, the N protein is an immunodominant protein that is linked to viral control in SARS-CoV-2 infection as it can elicit a robust T-cell response. The highly conserved N protein has been proposed as a potential immunogen for SARS-CoV-2 vaccination. The relevant experiments and specific outcomes of using the N protein as an immunogen for SARS-CoV-2 vaccination are displayed in Table 4.

**TABLE 4:** The research progress of SARS-CoV-2 nucleocapsid protein vaccine.

Vaccine name	Team	Vaccine type	Vector	Administration route	Dose	Animal experiment	Challenge trials	References
Tri: ChAd	Sam Afkhami	Viral Vectored Vaccine	ChAd	IM, IN	1	Mouse	B.1.1.7, B.1.351	Afkhami et al., 2022
Ad5-N	Tanushree Dangi	Viral Vectored Vaccine	Ad	IM	1	Mouse	WA1/2020	Dangi et al., 2021
Ad5-N	Jia He	Viral Vectored Vaccine	Ad	IN	2	Mouse	-	He et al., 2021
COH04S1	Flavia Chiuppesi	Viral Vectored Vaccine	MVA	IM, IN	1, 2	Hamster, NHP	WA1/2020	Chiuppesi et al., 2022
MVA/SdFCS-N	Nanda Kishore Routhu	Viral Vectored Vaccine	MVA	IM, BU, SL	2	Rhesus macaque, Mouse	WA-1/2020, β, delta (B.1.617.2)	Routhu et al., 2022
rACAM2000SN	YvonDeschambault	Viral Vectored Vaccine	VACV	IM	1	Hamster	ON-VIDO-01/2020	Deschambault et al., 2022
mRNA-S + N	Renee L. Hajnik	RNA vaccine	LNPs	IM, IN	2	Mouse, Hamster	MA-SARS-CoV-2, Delta, and Omicron	Hajnik et al., 2022
SpiN	Julia T. Castro	Recombinant Protein Vaccine	-	IM	2	Mouse, Hamster	BRA/SP02/2020, Delta, and Omicron	Castro et al., 2022
RBD-P2/N	So-Hee Hong	Recombinant Protein Vaccine	-	IM	2	Mouse, Rat, NHP	SARS-CoV-2	Hong et al., 2021

MVA, modified vaccinia Ankara; VACV, vaccinia virus; Ad, adenovirus; LNPs, lipid nanoparticles; IM, intramuscular; IN, intranasal; BU, buccal; SL, sublingual; NHP, non-human primate; Challenge trials: This column is the strain information of the challenge trials.

### 5.1. Theoretical basis for N protein as a promising vaccine target

So far, widely deployed COVID-19 vaccines have exclusively utilized the spike protein as the vaccine antigen. While these vaccines have effectively managed the COVID-19 pandemic, SARS-CoV-2 variants have emerged, characterized by mutations predominantly located within the immunodominant epitopes of the spike protein’s receptor-binding domain (RBD). This evolutionary process may result in the virus evading immune recognition, particularly by neutralizing antibodies (Maghsood et al., 2023; Murray et al., 2023). Although neutralizing antibodies impede the initial viral entry, T-cell responses play a pivotal role in controlling the infection and mitigating further viral dissemination in cases where the virus evades neutralizing antibody responses (Dangi et al., 2021). There is substantial evidence suggesting that the virus can propagate through direct cell-to-cell contact, which displays resistance to neutralizing antibodies (Zeng et al., 2022). This mechanism has been observed in various other viruses, underscoring the critical significance of T-cell immunity in viral elimination (Igakura et al., 2003; Moss, 2022). Notably, the N protein encompasses essential T-cell epitopes crucial for SARS-CoV-2 immunity (Le Bert et al., 2020; Lee E. et al., 2021; Lineburg et al., 2021; Khan et al., 2022). Recent studies have demonstrated that T cells specific to the NP _105–113_ -B*07:02 epitope can effectively recognize cells infected with both actively replicating SARS-CoV-2 virus and emerging viral variants, thereby exerting a substantial inhibitory effect on viral replication within infected cells (Peng et al., 2022).

The effects of cross-reactivity on SARS-CoV-2 infection and vaccination outcomes remain uncertain (Murray et al., 2023), and further research will contribute to the development of pan-coronavirus vaccines incorporating the N protein. Seasonally prevalent among the global population, the common cold coronavirus (CCC) accounted for approximately 10–20% of viral respiratory infections in 2019, predominantly manifesting as mild symptoms (Nickbakhsh et al., 2020). Given its high prevalence and substantial genetic resemblance to SARS-CoV-2, the CCC likely serves as a source of cross-reactive immune responses to SARS-CoV-2. Cross-reactivity regions between CCCs and SARS-CoV-2 reside outside the RBD, a region with minimal changes across SARS-CoV-2 variants. The research team led by Murray et al. (2023) indicates that cross-reactive immune responses resulting from previous CCC infections can profoundly influence the outcomes of SARS-CoV-2 infection and vaccination. Furthermore, pre-existing cross-reactive T cells may provide partial protection against COVID-19. Aran et al.’s (2020) research team found that individuals with a history of previous positive CCC tests exhibited decreased severity of COVID-19 after SARS-CoV-2 infection. This finding suggests the presence of cross-protection conferred by prior CCC infections.

Recent studies have demonstrated that the N protein plays a pivotal role in the innate immune system by stimulating T cell responses through FcR activation and participating in phagocytosis during natural infections (López-Muñoz et al., 2022; Maghsood et al., 2023). Literature suggests that COVID-19 vaccines incorporating S1 and N have the potential to combine neutralizing antibody responses against S1 with conserved T cell responses against N. Incorporating the N protein in vaccine formulations to enhance CD8^+^ T cell responses not only introduces more conserved regions of SARS-CoV-2 into the immune system to address potential emerging variants but also aids in viral clearance (Westmeier et al., 2020; Bange et al., 2021; Rha and Shin, 2021; Khan et al., 2022). The N protein, either alone or in combination with other SARS-CoV-2 antigens, can be regarded as an indispensable component for the design of SARS-CoV-2 vaccines.

Limited research was conducted on SARS-CoV-2 during the initial phases of the pandemic. In order to curtail the rapid transmission of this highly contagious and lethal virus, countries implemented a range of measures such as controlling epidemic areas and administering vaccines, aimed at preventing infections. Spike protein-specific antibodies play a critical role in preventing initial infections by spatially obstructing the interaction between the spike protein and the host receptor angiotensin-converting enzyme 2 (ACE2) (Lee E. et al., 2021). Over time, countries started implementing relaxation strategies, while new variants with immune evasion capabilities emerged to a certain extent. This considerably diminished the efficacy of previously administered vaccines, resulting in a persistent rise in infection cases. While N protein-specific polyclonal and monoclonal antibodies lack neutralizing activity and the ability to inhibit virus entry into human cells, they can stimulate CD8^+^ T cell responses, which play a protective role in situations of low or diminished antibody levels (McMahan et al., 2021; Moss, 2022). Given the considerable conservation of the N protein across various variants, its inclusion in vaccine formulations to induce robust CD8^+^ T cell responses can assist in managing infections and mitigating the severity of post-infection outcomes, even though it does not prevent infections caused by new viral strains evading the S protein (Peng et al., 2022). N protein could become an alternative to the currently popular vaccinal major target, the transmembrane spike protein. It is critical especially as new viral variants continue emerging even in the post-pandemic era.

### 5.2. Using N protein alone as a vaccine target

Although the use of N protein alone as a vaccine target may not provide protection to the vaccinated individual through the generation of high titers of neutralizing antibodies, it appears to confer protection through T cell-mediated immunity. The SARS-CoV-2 N protein contains several peptides that bind to human leukocyte antigen (HLA) CD4^+^ and CD8^+^ T-cell epitopes (Chen et al., 2020). According to some studies, the SARS-CoV-2 N protein might effectively trigger T-cell responses (Hong et al., 2021), which are essential for protection against primary SARS-CoV-2 infection (Sette and Crotty, 2021). In contrast, when Spike was not present in the trial, the production of the nucleocapsid protein did not elicit robust serum-neutralizing antibody responses (Buchholz et al., 2004). Therefore, vaccines targeting the N protein alone are not particularly protective.

Nasal washes from Hajnik et al.’s (2022) team experiment showed no significant difference in viral load between the mRNA-N vaccinated and placebo groups. The results suggest that mRNA-N alone is relatively ineffective in the upper respiratory tract due to the vaccine’s incapacity to generate neutralizing antibodies. Furthermore, the study demonstrated that intranasal vaccination was less efficient in controlling the virus in the lungs than intramuscular immunization. The failure of intranasal immunization with mRNA-N to provide protection is unsurprising given that it did not trigger an antibody response in the tested serum samples.

The application of the recombinant protein vaccine SpiN allowed Castro’s group to protect K18-ACE-2 mice from Delta and Omicron SARS-CoV-2 strains infection. Furthermore, the study demonstrated that T cells played a primary role in mediating SpiN’s protective immunity against SARS-CoV-2, rather than neutralizing antibodies (nAbs) (Castro et al., 2022).

### 5.3. Combining N protein with S protein can enhance the immunogenicity and protective efficacy of the vaccine

Afkhami et al. (2022) developed a trivalent vaccine, named Tri:ChAd, utilizing a chimpanzee adenovirus vector that contains S, N, and RdRp antigens. Intranasal administration of a single dose of this vaccine conferred protection against lethal infection by SARS-CoV-2 variants of concern (VOCs) and generated robust respiratory mucosal immunity not only to the ancestral SARS-CoV-2, but also reduced the immune evasion of VOCs in lethal infection. Challenge experiments showed that the vaccine was effective against B.1.1.7 and B.1.351 variants in mice. Dangi et al. (2021) combined spike-based and nucleocapsid-based vaccines using Ad5 as the vector. The study demonstrated that the combination vaccine induced spike-specific and nucleocapsid-specific antibody responses, as determined by ELISA. The titers for spike-specific antibodies exceeded 10^3^, while for nucleocapsid-specific antibodies, the titers exceeded 10^2^, providing evidence for the vaccine’s immunogenicity. The vaccine showed acute lung and brain protection compared to the S-based vaccine alone. However, co-immunization of mice did not confer any synergistic protective advantage in the lungs by day three post-challenge, suggesting that the spike-based vaccine alone was sufficient to protect the respiratory system. The research also indicated that nucleocapsid-specific immunity could improve distal control of SARS-CoV-2 and that such a vaccine protected mice from WA1/2020 challenge. He et al. (2021) also employed Ad5 as a vaccine vector. The nucleocapsid-based vaccine induced CD8 T cell responses in the lungs, while CD4 T cell responses were observed in the spleen, which was linked to elevated levels of antibody production. The antibody production was sufficient to protect mice against fatal SARS-CoV-2 infection and mitigate clinical symptoms.

Furthermore, MVA/SdFCS-N vaccine administered intramuscularly, generated cross-reactive antibody and T-cell responses against WA-1/2020 and δ strains in mice and macaques. Although the cross-neutralization activity against these VOCs was low, the vaccine provided complete protection against the β variant. Researchers identified a strong association between vaccine-induced neutralizing and non-neutralizing antibody effector activities and SARS-CoV-2 delta challenge protection. The initial vaccination with the vaccine induced similar levels of RBD-specific IgG antibodies and S-specific IgG antibodies in all vaccinated rhesus monkeys. The geometric mean titers for RBD and spike were approximately 6 × 10^3^ and 3 × 10^4^, respectively. At week 6 (2 weeks after the booster dose), the antibody titers increased by approximately 10-fold, with geometric mean titers of 5.8 × 10^4^ and 2.1 × 10^5^ for RBD and S, respectively, and remained stable at week 8. The vaccine-induced antibodies exhibited high neutralizing activity against the homologous (WA-1/2020) live virus, with a 50% neutralization titer reaching as high as 1,228 at 2 weeks post-vaccination (Routhu et al., 2022). rACAM2000, a vaccine expressing spike and nucleocapsid proteins, provides effective protection against SARS-CoV-2 in hamsters with a single intramuscular dose. Vaccination with rACAM2000S + rACAM2000N or rACAM2000SN reduces the viral load in the liver and small intestine more effectively than either S or N alone. rACAM2000N vaccination reduces virus levels more in distant tissues than in closer ones, indicating that the immune response elicited by rACAM2000N controls the spread of the virus beyond the initial site of infection (i.e., the lung). While rACAM2000 vaccination leads to higher neutralizing antibody titers following SARS-CoV-2 challenge, rACAM2000N vaccination does not. This finding suggests that cell-mediated immune responses are likely responsible for the protection afforded by rACAM2000N (Deschambault et al., 2022).

In comparison to the mRNA-S vaccine alone, the mRNA-S + N combination vaccine offers enhanced protection against variants of SARS-CoV-2 in both mice and hamsters. Additionally, this vaccine is capable of providing protection against the Delta and Omicron variants of SARS-CoV-2. The mRNA-S + N combination induces stronger suppression of the virus, decreasing viral RNA copies by an additional factor of 12, with mRNA-S reduced by a factor of 57, whereas mRNA-S + N reduced it by 770 times (*p*-value < 0.05). Furthermore, this combination vaccine demonstrates a higher efficacy in restricting lung infections from the Delta and Omicron variants and improving protection in the upper respiratory system (Hajnik et al., 2022). Previous studies of adenovirus vector vaccines have demonstrated that subunits of the N protein bound to the S protein enhance the immune response and provide greater protection compared to the S protein alone (Dangi et al., 2021). The reason for this might be that the N protein-specific immune response cannot prevent the virus from entering cells but can eliminate virus-infected cells. However, further investigation is necessary to determine whether the N protein is a valuable target for vaccines that can aid in the prevention or treatment of the disease.

## 6. Discussion

This review emphasizes the significance of utilizing multiple testing methods to monitor virus-specific antibody and antigen levels for evaluating disease status, risk of reinfection, and effectiveness of vaccine-induced immunity.

Most serological and nucleic acid tests target the N protein due to its stability. Measuring virus-specific antibody and antigen levels is not only fast and cost-effective, but the results can also be used to assess disease status, likelihood of reinfection, and duration of vaccine-induced immune responses (Zhao et al., 2020; Heyming et al., 2021; Rak et al., 2022; Uysal et al., 2022; Wei et al., 2022). One study revealed that elevated plasma N antigen levels correlated significantly with lung disease severity and could indicate systemic viral replication (Rogers et al., 2022). Currently, with the adjustment of China’s epidemic prevention policy at the end of 2022, more ordinary consumers are using rapid antigen detection (RAD) to determine whether they have been infected without leaving home, making a significant contribution to epidemic detection. However, rapid diagnostic tests or non-sequencing methods often cannot detect the specific strain of a patient’s infection, making it challenging for local CDCs to discover and track new variants. Additionally, rapid diagnostic tests generally cannot effectively quantify the viral load, limiting the usefulness of the results in predicting the course of the disease. Therefore, developing a kit that can identify virus strains and accurately quantify viral titer would greatly facilitate epidemic management and control. Data collation indicates that the detection sensitivity of various RAD kits for Omicron has decreased, particularly in samples with a low viral load (Ct ≥ 25). Thus, timely promotion of various detection kits for new variant detection is particularly important.

The COVID-19 pandemic has presented an ongoing challenge due to the emergence of variants of concern (VOCs) and the waning immunity induced by first-generation vaccines. Immune evasion by BQ and XBB variants has reached alarming levels, and earlier vaccines may not be effective against new variants (Wang et al., 2023). Therefore, there is an urgent need for new COVID-19 vaccines. The nucleocapsid protein shows promise as a universal vaccine target, as it can enhance immune defenses against current and future SARS-CoV-2 virus variants, as well as other coronaviruses that share high similarity with SARS-CoV-2, such as MERS and SARS. Developing vaccines targeting the N protein can reduce the cost and time needed for new vaccine development and enable more effective control and response to the pandemic. Although vaccines targeting the N protein alone may not provide adequate protection, combining them with the S protein can be more effective than targeting the S protein alone (Dangi et al., 2021). Future research should explore whether the N protein can effectively synergize with the S protein to enhance immunity.

## 7. Conclusion

In conclusion, it is imperative for the global community to continue investing in innovative research aimed at identifying and developing effective measures to end the COVID-19 pandemic. Sustained efforts are necessary to overcome this global public health crisis and establish a more resilient and prepared global health system.

## Author contributions

YZ, KC, and JHL conceived the manuscript. WS, ZF, and FM drafted the manuscript. WS, ZF, FM, JXL, and ZH performed the statistical analyses, interpreted the data, and generated figures. All authors contributed substantially to the content and reviewed or edited and approved the manuscript.
